# Spatio-temporal behaviour of atomic-scale tribo-ceramic films in adaptive surface engineered nano-materials

**DOI:** 10.1038/srep08780

**Published:** 2015-03-05

**Authors:** G. Fox-Rabinovich, A. Kovalev, S. Veldhuis, K. Yamamoto, J. L. Endrino, I. S. Gershman, A. Rashkovskiy, M. H. Aguirre, D. L. Wainstein

**Affiliations:** 1 Department of Mechanical Engineering, McMaster University, 1280 Main St. W. Hamilton, Ontario L8S 4L7, Canada; 2 Surface Phenomena Research Group, CNIICHERMET, 9/23, 2-nd Baumanskaya Street, Moscow 105005, Russia; 3 Materials Research Laboratory, Kobe Steel Ltd, 1-5-5 Takatsuda-dai, Nishi-ku, Kobe, Hyogo 651-2271, Japan; 4 Surface Engineering and Nanotechnology Institute (SENTi), School of Aerospace, Transport, and Manufacturing, Cranfield University, College Rd, Cranfield, Bedford MK43 0AL, United Kingdom; 5 All-Russian Railway Research Institute, 10 Third Mytishchinskaya Street, Moscow 29851, Russia; 6 National University of Science and Technology “MISiS”, Leninskiy prosp. 4, Moscow 119049, Russian Federation; 7 Laboratory of Advanced Microscopy, Insitute of Nanoscience of Aragón, University of Zaragoza, 50018 Zaragoza, Spain

## Abstract

Atomic-scale, tribo-ceramic films associated with dissipative structures formation are discovered under extreme frictional conditions which trigger self-organization. For the first time, we present an actual image of meta-stable protective tribo-ceramics within thicknesses of a few atomic layers. A mullite and sapphire structure predominates in these phases. They act as thermal barriers with an amazing energy soaking/dissipating capacity. Less protective tribo-films cannot sustain in these severe conditions and rapidly wear out. Therefore, a functional hierarchy is established. The created tribo-films act in synergy, striving to better adapt themselves to external stimuli. Under a highly complex structure and non-equilibrium state, the upcoming generation of adaptive surface engineered nano-multilayer materials behaves like intelligent systems - capable of generating, with unprecedented efficiency, the necessary tribo-films to endure an increasingly severe environment.

Introducing dissipative non-equilibrium processes constitutes one of the major goals and challenges for future developments in modern material science^1^,^2^. The way to achieve this goal is through constructing dynamic artificial systems which can adapt to their environment through the formation of hierarchical spatiotemporal structures^3^.

The mechanism of adaptation of non-equilibrium natural processes is self-organization with dissipative structures formation^4^,^5^. In engineering systems, similar behaviour is typical in particular for tribo-systems through the dynamic generation of tribo-films caused by tribo-chemical reactions on the friction surface as a result of interaction with the environment^5^. The ordering power of self-organization could be fully demonstrated for systems interacting with a far from equilibrium extreme environment, close to the ‘edge of chaos’^6^. For instance, the ultra-speed dry machining of hardened tool steels is a somewhat unique case of extreme tribo-conditions where an adaptive system can exhibit its full potential. Under such conditions, high temperatures (1000–1200°C) and heavy loads (3–5 GPa) develop on the friction surface of coated cutting tools^3^. An upcoming generation of surface engineered nano-materials is reported, represented by hard adaptive nano-multilayer TiAlCrSiYN-based Physical Vapor Deposited (PVD) multilayer coatings specifically designed for extreme frictional conditions^3^,^7^,^8^. It exhibits multifunctional adaptive behaviour and very high wear resistance under outlined tribological conditions through its enhanced ability to form thermal barrier/lubricating tribo-films on the friction surface^3^.

Previously published experimental observations showed that non-equilibrium films drastically affect the heat transfer in the cutting area, strongly localizing the heating zone, which results in accumulation of friction generated heat, its dissipation via various channels (Ref. 3) and enormous (estimated around 400–600°C^9^) temperature gradients within the layer of tribo-films on the friction surface. However, the important features of the spatiotemporal behaviour of these dynamic non-equilibrium phases forming on the surface have remained unknown.

In this paper, we report the formation of spatiotemporal atomic-scale dynamic structures on the friction surface with amazing energy soaking/dissipating ability. The tribo-film layers exhibit multi-functional and synergistic performance in response to an intensifying environment^10^,^11^. While adapting to an extreme environment, the layer of atomic-scale dynamic tribo-films control overall system performance^10^,^11^ and exhibit hierarchical behavior^12^,^13^,^14^,^15^.

## Results and Discussion

### Structure of the coating in as-deposited state

The coating layer plays a critical role in providing a stable environment for the system to display adaptive behavior. Rapid destruction of hard and brittle tribo-ceramics could be prevented with the proper design of the coating layer that can provide a low wear and surface damage resistant environment for the tribo-films to form in order to sustain high temperatures/stresses under operation. In this way, they can efficiently act as thermal barriers and in response, protect the underlying coating layer^7^,^9^. The stability of the self-organization process could be controlled by dynamic self-regulation of the tribo-film generation and destruction^11^.

**Figure 1:**
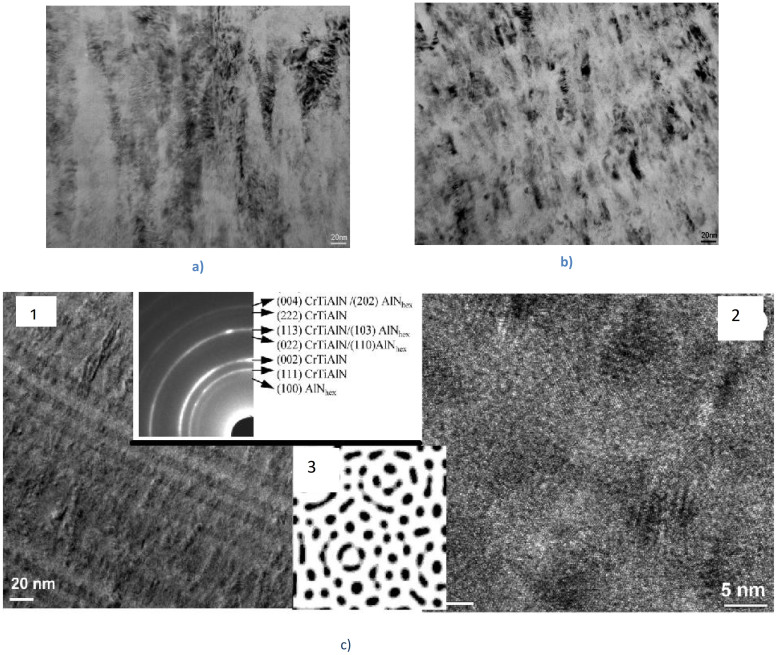
The TEM cross-sections of TiAlCrSiYN-based coatings: (a) TiAlCrSiYN monolayer coating; (b) TiAlCrSiYN/TiAlCrN multilayer coating; (c) the TEM cross-section of TiAlCrSiYN/TiAlCrN coating in as-deposited condition: 1- the structure of interfaces is uniform; the nano-layers have columnar structure; the variable contrast 2- is consequence of modulation of chemical composition and strains in a matrix; 3- “drop-like” decomposition mechanism of (TiAlCr)N layer in presence of a powerful drain of vacancies^5^.

This could be realized in the multilayer coating in terms of its structure and properties^7^,^9^,^17^. Structures of the mono- and multilayer TiAlCrSiYN-based coatings are presented in Figure 1. It was shown that the monolayer coating has a nano-crystalline structure (Figure 1, a)^16^ and the multilayer coating has a more complex structure that combines the nano-multilyer structure with a modulating composition and a columnar nano-structure (Figure 1, b)^17^. Moreover HREELS studies confirm that the multilayer coating possesses a higher non-equilibrium state than the monolayer^8^.

**Figure 2:**
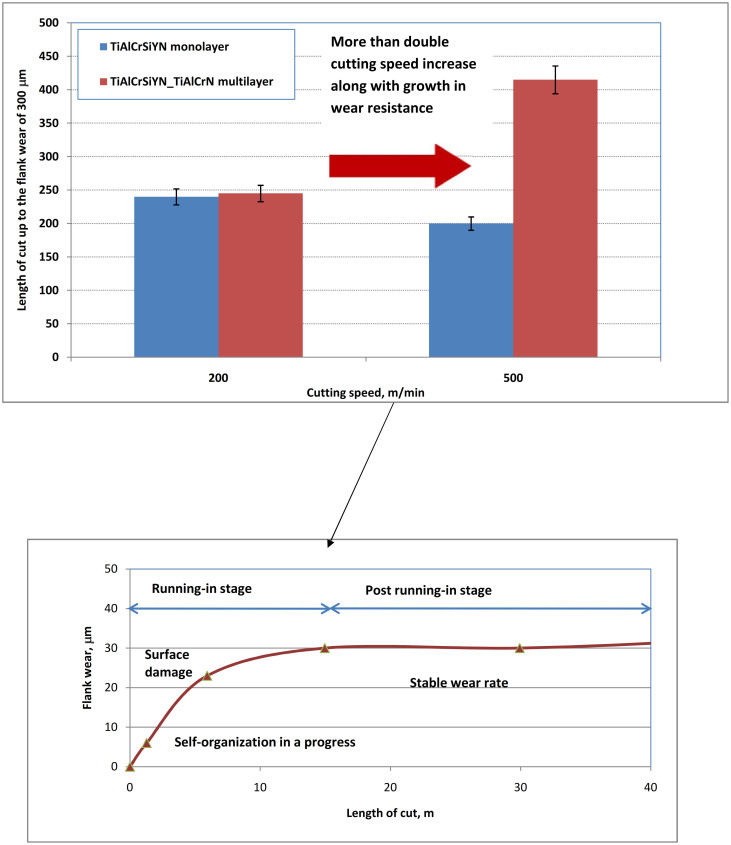
Wear resistance of adaptive surface engineered nano-materials: (a) wear rates of monolayer vs. multilayer; (b) typical stages of wear process.

There is other important evidence of the non-equilibrium structure of the multilayer coating strongly affecting tribo-chemical reactivity of the coating layer. As it is shown in Figure 1, c-1 multilayer coating is formed as a result of epitaxial growth and has clearly visible inter-grain boundaries. A small amount of hexagonal AlN phase is found in the TiAlCrN nano-layers. The differences in contrast presented in Figure 1, c-2 are due to the formation of chemical heterogeneity by chromium, which is typical for spinodal decomposition^18^,^19^.

Under specific conditions, the formation of the concentration modulation in solid phase composition of thermodynamically unstable solid solutions can be energetically more favorable than the de-composition into separate phases^20^. Periodic spatial distribution of concentration within a certain temperature range occurs when the coefficients of mutual diffusion, self-diffusion of the elements in a complex solid solution are positive and close in value. In this case, the multi-component system becomes unstable to the diffusion, and it is prone to the occurrence of the modulated structures during the processes of growth of the coating layer.

**Figure 3:**
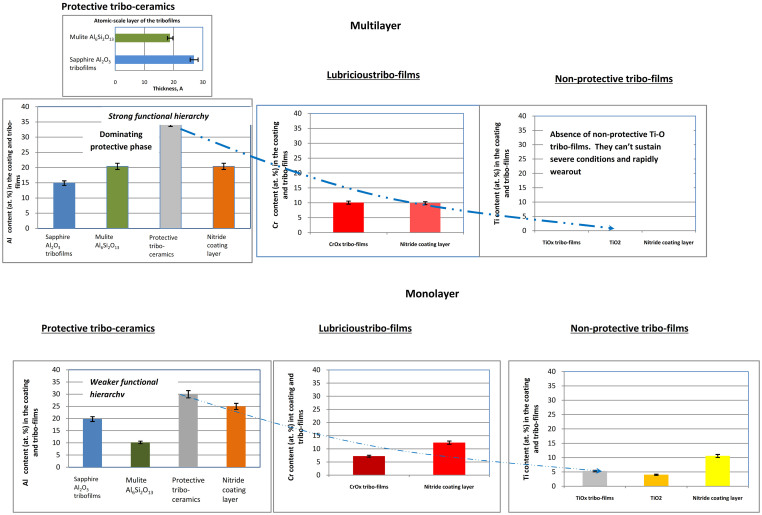
Comparison of tribo-film phase composition on the worn surface of multilayer and monolayer nano-materials during running-in stage (length of cut of 15 m). (XPS data, see initial spectra in supplementary information). The dashed lines show trends to guide the eye only.

HRTEM (Figure 1, c-1) demonstrates the columnar nano-grains of TiAlCrSiYN and TiAlCrN. Contrast modulation in Figure 1, c-2 is more related to the de-composition of the solid solution and the emergence of the “drop-wise” nano-regions with a cubic nitride structure, enriched and depleted by chromium. The microstructure of the spinodal decomposition is presented in Figure 1, c-3. It shows typical pattern of the surface directed spinodal decomposition (SDSD) with disturbance of thermodynamic parameters near the boundaries.

Analysis of numerous theoretical models of spinodal de-composition gives us the possibility of suggesting a scheme of formation for the observed structures^21^,^22^,^23^. Most likely, the main cause of the de-composition is related to the coatings' crystallization process during deposition. The columnar-like structure of the coating is characterized by the considerable disorientation of the nano-crystals and high concentration of “frozen” vacancies. It was shown that phase stability is significantly influenced by non-equilibrium nano-grained alloys with grain boundaries causing lattice distortions in adjacent regions^23^. The kinetic decomposition depends on the deviation of the equilibrium conditions near grain boundary from bulk values. Angle grain boundaries are powerful sources and sinks of vacancies and atoms. We have to realize that the outer surface of the thin film coating and the interfaces are also the sources and sinks of vacancies. Diffusion flux of vacancies from the bulk to the sinks are enhanced by cooling and crystallization of the alloy. The segregation of one of the coating's components will locally alter the concentration in the subcritical region of the phase diagram and stimulate the phase separation in the alloy, which is stable in coarse grained material. Even if segregation is neglected (i.e. the deviation of the average composition near the grain boundaries from bulk is small), the increase of the mixing energy in the grain boundary area will change character of the transformation to the spinodal decomposition and give rise to concentration oscillations along the grain boundary. Figure 1, c-3 illustrates the development of SDSD with forming of zones poor and rich in Cr. The occurrence of segregations determines further transformation kinetics and as a result, the ordered “drop-wise” domain structure in concentrated solid solution is realized.

Thus, in both cases, the de-composition of the meta-stable nitrides is accelerated due to the high concentration of vacancies, which is typical for the PVD coatings due to the ion bombardment during their synthesis. It was found that the grain boundaries can stimulate the appearance of concentration waves propagating inside the nanograins when homogeneous alloy state is unstable with respect to spinodal decomposition^23^,^24^.

The structural state of the as-deposited coating is fairly complex. On one hand, the spinodal decomposition of the deposited coatings implies some degree of their transformation to a temporary steady state. On the other hand, the formation of micro-chemical heterogeneity during the synthesis of a multilayer coating is accompanied by the formation of high density of “frozen” vacancies. The rresultant structure is very non-equilibrium and helps to intensify the mass transfer during the very initial stages of the tribo-oxidation. We think this could explain the high tribo-chemical activity of the multilayer nitride coating during the running-in stage of wear.

We have to note that a highly non-equilibrium state of nano-materials could be considered as an indicator of their temporal behavior. This generic characteristic provides the system with strongly accelerated rate of physical-chemical reactions on the friction surface associated with the mobility and reactivity of the coating material elements. Moreover the coating layer is capable of displaying intelligent behaviour under operation.

### Tribological performance

Wear behaviour of the TiAlCrSiYN monolayer vs. TiAlCrSiYN/TiAlCrN multilayer is shown in Figure 2. The monolayer demonstrates a predictable trend, typical for the majority of wear resistant PVD coatings of the TiAlN family used for extreme tribological applications^5^: wear resistance noticeably decreases with growing severity of external stimuli (in particular due to the cutting speed growth, Figure 2). In contrast, the multilayer exhibits a quite opposite trend. An unusual feature of the multilayer material is its ability to increase its wear resistance substantially after a cutting speed raise from a commonly practical speed of 200 m/min^5^ up to ultra high speed conditions of 500 m/min. Wear curves representing wear rate vs. length of cut are presented. It has been shown that self-organization during friction is developing within the initial, most harsh running-in stage^5^,^8^, which chiefly determines future wear behavior^5^,^8^,^16^. To explain the cause of such unusual performance, the adaptive behaviour of the nanomultilayer material was studied in the tribofilm formation during the running-in stage of wear (after length of cut of 15 m). To better understand these phenomena, we related them to similar data for the previous generation of wear resistant materials (TiAlCrSiYN monolayer)^16^,^17^.

### Surface analysis

XPS studies of the surface layer allow us to determine the chemical and phase composition of the wear products that are the most stable and remain on the surface during the running-in stage (Figure 3, also see initial spectra in ‘XPS data’ in supplementary information). A major feature of the studied adaptive coatings is the ability to form atomic-scale layer of the tribo-ceramics with extremely high protective ability (Figure 3; supplementantary information)^25^,^26^. This radically changes the thermal properties on the friction surface.

The major difference in wear behavior of mono - and multilayer materials under extreme tribological conditions lies in the accelerated beneficial tribo-oxidation on the surface of the multilayer (Figure 3). A substantially greater amount (almost twice) of mullite tribo-films is observed forming on the worn surface of the multilayer compared with the monolayer. In addition, there is no indication of Ti-O or presence of initial Ti-N phases on the surface of the multilayer. This means intensive tribo-oxidation of the titanium-based phases is taking place, leading up to these entire compounds selectively wearing out from the friction surface (Figure 3). Therefore, only protective tribo-oxides of aluminum, lubricating oxides of chromium, and even small amount of beneficial Y_2_O_3_ films (see ‘XPS data’ in supplementary information) actively form on the surface of the multilayer material, reducing wear rate (Figure 2). In this way our artificially intelligent tribo-system ‘selects’ the necessary surface structures that it requires to sustain in a given external environment. The overall amount of protective phases (sapphire and mullite) is higher than in the monolayer material (Figure 3) and highly protective thermal barrier tribo-ceramics begin to predominantly form Ref. 8. They establish a strong functional hierarchy in the tribo film layer through selective adaptation to the external stimuli^8^,^12^,^13^. In contrast, the amount of the initial nitride phase in a monolayer is 1.6 times higher compared to the multilayer, and therefore, its surface protection/lubrication is less efficient.

**Figure 4:**
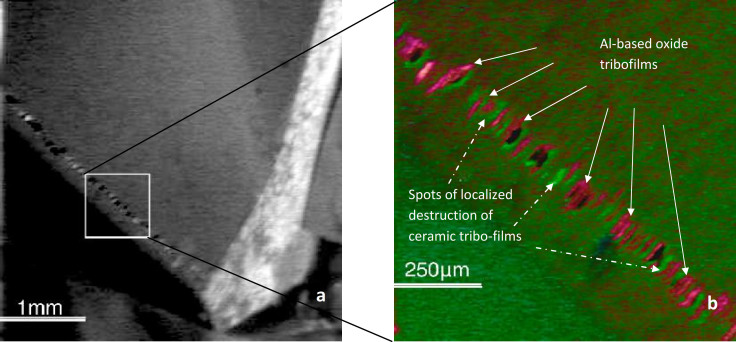
Auger image of atomic scale tribo-films forming on worn surface (a) – general image of in O KLL_512_ (topography contrast); (b) – red image in O KLL_508.5_; light green image in N KLL_377.5_.

Employing Auger imaging we are able to exhibit “snap shots” of the dynamically re-generating atomic scale surface films as a result of the series of tribo-chemical reactions on the friction surface (Figure 3 and Figure 4). To the best of our knowledge this is the first actual image of dynamic meta-stable phases directly associated with the dissipative structures (as a process) that form in far from equilibrium tribological conditions. Figure 4 shows the scanning Auger image of the worn surface. The general view of the worn area is shown in Figure 4, a. Detailed distribution pattern of aluminum-based tribo-oxides and non-stoichiometric nitride within the worn area is shown in Figure 4, b. The resulting Auger image represents the meta-stable phase distribution within the worn area.

This allows us to re-construct the temporal behavior of the tribo-films. Atomic-scale films of aluminum-based oxides (presented by red spots) are forming (Figure 3; 4, b), segregated by the areas of non-stoichiometric nitride phase (green spots), which are laid openly due to the localized destruction of ceramic tribo-films. A periodic pattern of the re-generating process is clearly observed, indicating its ordering behavior. It is worth noting that after wearing out, a new portion of the the tribo-films will continuously form. This is also the cause of their high efficiency in surface protection.

**Figure 5:**
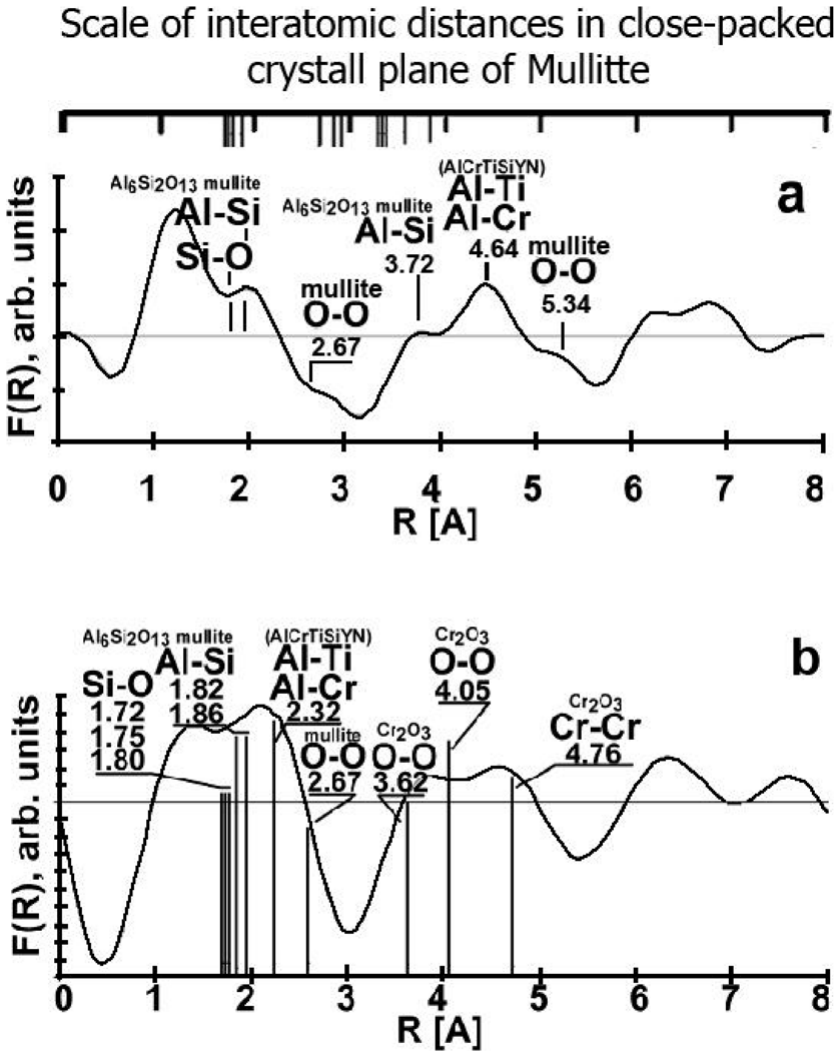
Fourier transform of EELFS from tribo-oxides formed on worn surface of multilayer nano-material: (a) during running-in stage; (b) during post running-in stage.

We have to take into account the real structure of the tribo-oxides. In fact, they have very complex atomic-crystalline structures^3^ representing dynamic state of complex matter^2^ (Figure 5). Extremely thin films of tribo oxides have a nanocrystalline structure with different degrees of perfection. Studies of the atomic structure of Al-O films formed on the surface of the TiAlCrSiYN-based surface engineered material, showed that they mostly have a short-range order, closest to the structure of mullite or sapphire only within the first coordination spheres (representing near-amorphous non-stoichiometric oxygen-containing phases) but some amount of crystalline phase has been also found^3^,^5^,^9^,^17^ (Figure 5). This is related to XPS data indicating crystalline phase formation as well (Figure 3 and XPS data in supplementary information).

Evolution of the tribo-film atomic structure during different stages of wear can be evaluated by comparing Figures 5, a and b. It is seen that the peaks in Figure 5, a are sharper and more pronounced. This indicates greater perfection of the crystal lattice of the thinnest (18.8 Å thick, Figure 3, supplementary information) mullite films formed during running-in stage of wear (length of cut of 15 m).

Mullite has a noticeably lower thermal conductivity than sapphire and, therefore, thermal protection goes up once large portions of the crystalline mullite phase had formed^26^,^27^,^28^. Thus, an increased amount of crystalline mullite (Figure 3, 5) films are formed, indicating enhanced adaptive performance and hierarchical behaviour of the tribo-film layer in response to the harsh conditions of the running-in stage. It should be noticed that the thermal conductivity of amorphous-like materials, with their lack of a crystalline order, is also low^28^. This was attributed to their smaller crystal size, and strong phonon scattering at the poorly interconnected nano-crystal boundaries (boundary scattering)^29^. Altogether these factors result in enormously high thermal barrier properties developing within the film layer at the atomic scale.

In amorphous-like tribo-films, the material may expose super-plasticity^5^,^30^. This is especially true for the alumina-based films doped by Y_2_O_3_ phase^30^ (see corresponding XPS spectra in supplementary information). Tribo-films of this type, in conjunction with lubricating chromium oxides, promote energy dissipation during friction^5^. This is a fascinating case of their multi-functional, synergistic behaviour.

## Discussion

We demonstrated that multilayer TiAlCrSiYN/TiAlCrN thin film PVD coating is an example of the upcoming generation of adaptive wear resistant surface engineered nano-materials which possess initial compositional and structural characteristics complex enough to develop via the cooperative interaction of its components in response to an intensifying external environment. Over time, it virtually transforms into a system with a greater complexity and spatio-temporal, integrative performance. The adaptive surface engineered nano-material exhibits intelligent behaviour with the ability to self-protect its surface.

Dynamically forming tribo-films play a critical role in the adaptive performance of the whole system, since the entirety of their actions are aimed to reduce wear rate to the lowest possible.

We can outline a number of important features in the tribo-film behavior:

- Atomic scale phenomena (spatial miniaturization);
- Ability of the surface engineered system to form a new series of tribofilms through interaction with the environment occurring as soon as the old series is worn out;
- Establishment of hierarchical behaviour (predominating formation of most efficient mullite tribo-phases) within the atomic-scale layer of the tribo-films due to selective adaptation to the environment.
- A strong interconnection between various tribo-ceramic components optimizing complementary possibilities and functions (synergistic behaviour). In this way, the entire capacity of the system is employed for adaptation to the extreme environment with greatest possible efficiency.

Dynamic thin films exhibit enormous energy soaking/dissipating capacity at an atomic scale, resulting in the ability of the whole system to efficiently endure external stimuli. A more deep understanding of this process could give us, on one hand, the possibility to unravel the mechanisms behind the meta-stable phases behavior and on the other hand, develop methods to control them, in order to enhance the capabilities of complex matter.

Realizing the full extent that self-organization plays at atomic scale spatiotemporal structures, may bring forward a new approach to nano-science and technology which might result in breakthrough developments in new generations of nano-materials with an enormous adaptive potential.

## Methods

Two different coatings were compared, a TiAlCrSiYN monolayer and a TiAlCrSiYN/TiAlCrN multilayer. The monolayer Ti_0.2_Al_0.55_Cr_0.2_Si_0.03_Y_0.02_N and nano-multilayered Ti_0.2_Al_0.55_Cr_0.2_Si_0.03_Y_0.02_N/Ti_0.25_Al_0.65_Cr_0.1_N coating was deposited using Ti_0.2_Al_0.55_Cr_0.2_Si_0.03_Y_0.02_ and Ti_0.25_Al_0.65_Cr_0.1_ targets correspondingly fabricated by a powder metallurgical process on a mirror polished cemented carbide WC-Co substrate and ball nose end mills in a laboratory type arc Plasma Vapour Deposition (PVD) coater (AIP SS002, Kobe Steel Ltd.) equipped with a plasma-enhanced cathode (Kobe Steel Ltd.) using a plasma-enhanced arc source. Samples were heated up to about 500°C and cleaned through Ar ion etching process. Ar–N mixture gas was fed to the chamber at a pressure of 2.7 Pa with a N_2_ partial pressure of 1.3 Pa. The arc source was operated at 100 A for a 100 mm diameter × 16 mm thick target. Other deposition parameters are as follows: bias voltage: 100 V; and substrate rotation: 5 rpm. The thickness of the coatings was around 3 microns for the film characterization and cutting test work.

The high-resolution transmission electron microscopy (HRTEM) and selected area electron diffraction (SAED) were performed by Tecnai F30 FEI microscope.

The phase composition and electronic structure of coatings were studied by X-ray photoelectron spectroscopy (XPS). Spectra were acquired by the ESCALAB MK2 (VG) electron spectrometer and processed by UNIFIT 2007 software. X-ray tube with monochromatic Al K_α_ radiation (hν = 1486.6 eV) was used as an excitation source. Accuracy of the data obtained by XPS was 10%.

Scanning Auger imaging of the worn area has been performed in characteristic emission of Auger electrons O KLL_512_ (a) and O KLL_508.5_, N KLL_377.5_ (b). Kinetic energies of O KLL (508.5 eV) and N KLL (377.5 eV) are accordance to Al_2_O_3_ and non-stoichiometric complex nitrides.

The atomic structure of the tribofilms was investigated using Electron Energy Losses Fine structure (EELFS) analysis^29^. The fine structure of electron losses spectra in the 250 eV range near back-scattered peak (E_0_ = 1500 eV) has been analyzed. The diameter of spot for investigated micro area was approximately of 100 μm. The fine structure of the electron spectra contains information about the structure of the nearest atomic neighbors on the surface. Mathematical spectra processing methods were used to analyze the fine structure of the electron spectra, which allows determining the lengths of the atomic bonds^31^.

The Fourier transforms (an analog to Radial Distribution Function^31^) are shown in Figure 5, a, b. The EELFS spectra were obtained from two different places at worn surface after length of cut of 15 (running-in stage of wear) and 30 m (post running-in stage of wear) correspondingly. Each peak position corresponds to the radius of coordination sphere in the crystal lattice. Interpretation of Fourier transforms was based on the data on phase structure (in accordance with XPS results, Figure 3 and supplementary information), and the standard crystallography data of interatomic distances in unit cell. In Figure 5 the standard interatomic distances of 1–3 coordination spheres are shown for the phases identified by XPS. The structural peaks in the Fourier transform in Figure 5, a correspond to the inter-atomic distances in the mullite-like aluminum-silicon oxide and nonequillibrium complex nitride^23^,^24^. The concentration of chromium oxide was small. For this reason, the peaks from chromium oxide are absent from the Fourier image of analyzing area. Distinct and easily distinguished peaks are observed on the Fourier transforms up to distances of the order of 7 Å. This means near and long-range order in the atomic surrounding is observed in these thin oxide films.

The Fourier transform in Figure 5, b was calculated for the other area at the worn surface after length of cut of 30 m. In this case, it has four wide peaks at 1.0–5.0 Å inter-atomic intervals (Figure 5, b). The first peak is non-structural. Complex chemical composition of the tribofilms provides a large set of paired inter-atomic distances. The phase with high concentration makes the biggest input in the radial distribution function. The first structural intense peak is formed by the mullite oxide and the non-oxidized coating (Figure 5, b). Two wide peaks at 3.5–5.0 A can be associated with imperfect chromium oxide Cr_2_O_3_. One can see in Figures 4, a–b that two phases, namely sapphire- and mullite-like tribofilms co-exist on the friction surface because the measured lengths of the nearest inter-atomic Al-O, O-O Si-O, Si-Si bonds are close to the standard crystallographic data of these phases (as shown on the scale in Figure 5). The broadening of the coordination spheres' peaks is taking place due to a high concentration of defects in the tribofilms.

Cutting tests have been performed during dry ball-nose end milling (Mitsubishi carbide ball nose end mills, D = 10 mm) of the hardened AISI H13 tool steel with hardness HRC 53–55. The cutting parameters were as following: speed 500 m/min; feed: 0.06 mm/tooth; axial depth: 5.0 mm; and radial depth: 0.6 mm. The coated tool flank wear was measured using an optical microscope (Mitutoyo model TM). A tool dynamometer (9255B, Kistler) was used to measure the cutting forces. At least three cutting tests were performed for each kind of coating. The scatter of the tool life measurements was approximately 10%.

## Supplementary Material

Supplementary Information Supplementary information
