# Supplementary material for: Spatio-temporal behaviour of atomic-scale tribo-ceramic films in adaptive surface engineered nano-materials
Source: Sci Rep. 2015 Mar 5;5:8780. doi: 10.1038/srep08780 (PMC5390086; doi:10.1038/srep08780)
Supplement: Supplementary Information [file srep08780-s1.pdf]

Supplementary information

Spatio-temporal behaviour of atomic-scale tribo-ceramic films in adaptive surface engineered nano-materials

G. Fox-Rabinovich, A. Kovalev, S. Veldhuis, K. Yamamoto, J. L. Endrino, I.S. Gershman, A. Rashkovskiy, M. H. Aguirre, D. L. Wainstein

XPS data on the tribo-films formation.

Figure 1 shows general photoelectron spectra from the worn area on the rake surface of the tool with mono- and multilayer TiAlCrSiYN-based coatings after length of cut 15 m (running-in stage of wear). By comparing these spectra, it is clear that the phase composition of the wear products differ significantly for the mono- and multilayered coatings. The amount of titanium oxide is significantly lower on the worn surface of the multilayer coating. In contrast, the amount of silicon and aluminum content in tribo-ceramics is much higher.

There are two lines of silicon and aluminum at different kinetic energies in these spectra. This gives us a unique opportunity to determine the thickness of the tribo-oxide film. Tribo-oxides, which form on the surface of worn tools, have complex phase and chemical composition. The dynamically re-generating films of simple and complex meta-stable tribo-oxides and spots of un-oxidized initial nitride phase are appearing on the worn surface. Under these conditions, the precise measurement of film thickness using the Electron Spectroscopy for Chemical Analysis (ESCA) method is difficult [1]. However, it is possible to make an estimate by measuring the divergence from the standard ratio of intensities of the photoelectron lines for corresponding mean free passes. A line of silicon and aluminum 2s and 2p appears on the photoelectron spectrum of the wear surface of the multilayer coating after cutting length of 15 m (Figure 1, a). Standard line intensity ratio of silicon (ISi2s/ISi2p) = 0.988. The

mean free pass for these lines is 30.75 and 31.70 A, respectively. Standard line intensity ratio of aluminum (IAl2s/IAl2p) = 1.188, experimental - 1.15. The mean free pass for these lines is 27.33 and 28.05 A, respectively. This means the thickness of the oxide film is somewhat less than 27.33-28.05 A. The experimentally obtained value of the ratio of silicon (ISi2s/ISi2p) = 0.383, which is almost three times lower than the standard photo-ionization cross sections corresponding to the thickness of the oxide film of the order of 18.8 A. We can attribute this thickness to the mullite tribo-ceramic films because XPS indicates strong Al-Si bonds typical for this tribo-ceramic. Low intensity of the silicon Si2s does not permit calculating the (ISi2s/ISi2p) ratio reliably for a cutting length of 30 m. For this reason, more intensive aluminum lines could be used to determine the thickness of the tribo-oxide film. The ratio of intensities of the lines (Al2s/Al2p) = 0.988 is less than 1.19 times the theoretical value (1.18). This leads to the conclusion that reduction in the intensity of low-energy line Al2s can take place if the oxide film thickness is of the order of 27.33 A. In both cases, the tribo-oxide films have a very small, atomic-scale thickness, which increases with cutting time (Figure 3 in the main text).

Figures 2-4 present detailed data on the phase composition of the worn surface of cutting tools with monolayer and multilayer coatings. Figures 2, a-b show the components of photoelectron lines Al2s, corresponding to the different types of chemical bonds of aluminum in simple and complex oxide films and nitride coatings. Previously, such meta-stable phases have been observed on the worn surface of the coated ball nose end mills within post-running stage of wear [2]. Chemical shifts of the Al2s component regulate the phase composition of aluminum-based compounds on the worn surface. Al-Si-O bonds generate in the composite oxide with the mullite –structure. Al-O bonds are typical of the Al_2O_3 composite and Al-N bonds correspond to the spots of the initial nitride coating, which have not been oxidized during cutting. Figure 2, c exhibits the difference in the tribo-chemical behavior of the multilayer and mono-layer coatings. For the multilayer coating, the content of mullite tribo-phase on the surface is increased while the amount of sapphire-like oxide and initial nitride is significantly lower compared to the monolayer. It can be seen by analyzing the chemical shift in O1s line (Figures 3, a-b) that

the products of wear in the multilayer coating contain only mullite, sapphire and chromium tribo-oxides. In contrast titanium oxide is detected on the worn surface of the monolayer coating.

Figure 4 shows the photoelectron spectra Si2s and $Y3d_3$ / 2 from the worn surface of the multilayer (a, b) and monolayer (c) coatings. Formation of Y oxides is detected. Small amount of stoichiometric Y_2O_3 oxide and non-equilibrium $Y_{1-x} O_x$ oxide are detected on the worn surface. With wear of the multilayer coating the amount of stoichiometric Y_2O_3 oxide grows vs. the non-equilibrium oxide. Reverse pattern is observed for the monolayer coating. The contents of these oxides are less than a fracture of at %. However we have to note Y_2O_3 has many beneficial properties, especially in conjunction with alumina, including: high chemical and thermodynamic stability; low thermal conductivity [3-5] as well as high temperature lubrication properties [6].

Fine structure analysis of photoelectron Si2s line shown in Figure 4 indicates the mullite tribo-films formation on the worn surface. Phase analysis of the tribo-films formed during the running-in stage (after length of cut of 15 m) is summarized in Figure 3 in the main text.

Reference:

1. Siegbahn, K. (1970). Electron spectroscopy for chemical analysis (ESCA). *Philosophical Transactions of the Royal Society of London. Series A, Mathematical and Physical Sciences*, *268*(1184), 33-57.
2. G. S. Fox-Rabinovich, K. Yamamoto, B.D. Beake, I. S. Gershman, A.I. Kovalev, M. H. Aguirre, S.C. Veldhuis, G. Dosbaeva, J. L. Endrino, Hierarchical adaptive nano-structured PVD coatings for extreme tribological applications: the quest for non-equilibrium states and emergent behavior, Sci. Technol. Adv. Mater. (STAM) 13 (2012) 043001
3. N. P. Padture, P. G. Klemens, Low Thermal Conductivity in Garnets, J. Am. Ceram. Soc., 80, [4] 1018–20 (1997)
4. N. P. Padture, P. G. Klemens, Low Thermal Conductivity in Garnets, J. Am. Ceram. Soc., 80, [4] 1018–20 (1997);
5. L. Sun, H. Guo, H. Peng, S. Gong, H. Xu, Phase stability and thermal conductivity of ytterbia and yttria co-doped zirconia, Progress in Natural Science: Materials International Volume 23, Issue 4, August 2013, Pages 440–445
6. Y. J. Su,R. W. Trice,K. T. Faber,H. Wangand W. D. Porter, Thermal Conductivity, Phase Stability, and Oxidation Resistance of Y3Al5O12 (YAG)/Y2O3–ZrO2 (YSZ) Thermal-Barrier Coatings, Oxidation of Metals, Vol. 61, 3/4, 2004, 253-271

Figure captions:

Figure 1 SI: General photoelectron spectra of worn surface of the multilayer TiAlCrSiYN/TiAlCrN – (a) and monolayer TiAlCrSiYN (b) coatings after length of cut of 15 m.

Figure 2 SI: Al2s photoelectron spectra of the worn surface of multilayer TiAlCrSiYN/TiAlCrN – (a) and monolayer TiAlCrSiYN (b) coatings within running-in stage of wear (after length of cut of 15 m); (c) – Differential (c)= (a)-(b) photoelectron spectrum.

Figure 3 SI: O1s photoelectron spectra of the worn surface of the multilayer TiAlCrSiYN/TiAlCrN – (a) and monolayer TiAlCrSiYN (b) coatings within running-in stage of wear (after length of cut of 15 m).

Figure 4 SI: Y3d and Si2s photoelectron spectra of worn surface of multilayer TiAlCrSiYN/TiAlCrN – (a, b) and monolayer TiAlCrSiYN (c) coatings within running-in stage of wear (after length of cut of 15 m).

Figure 1 SI

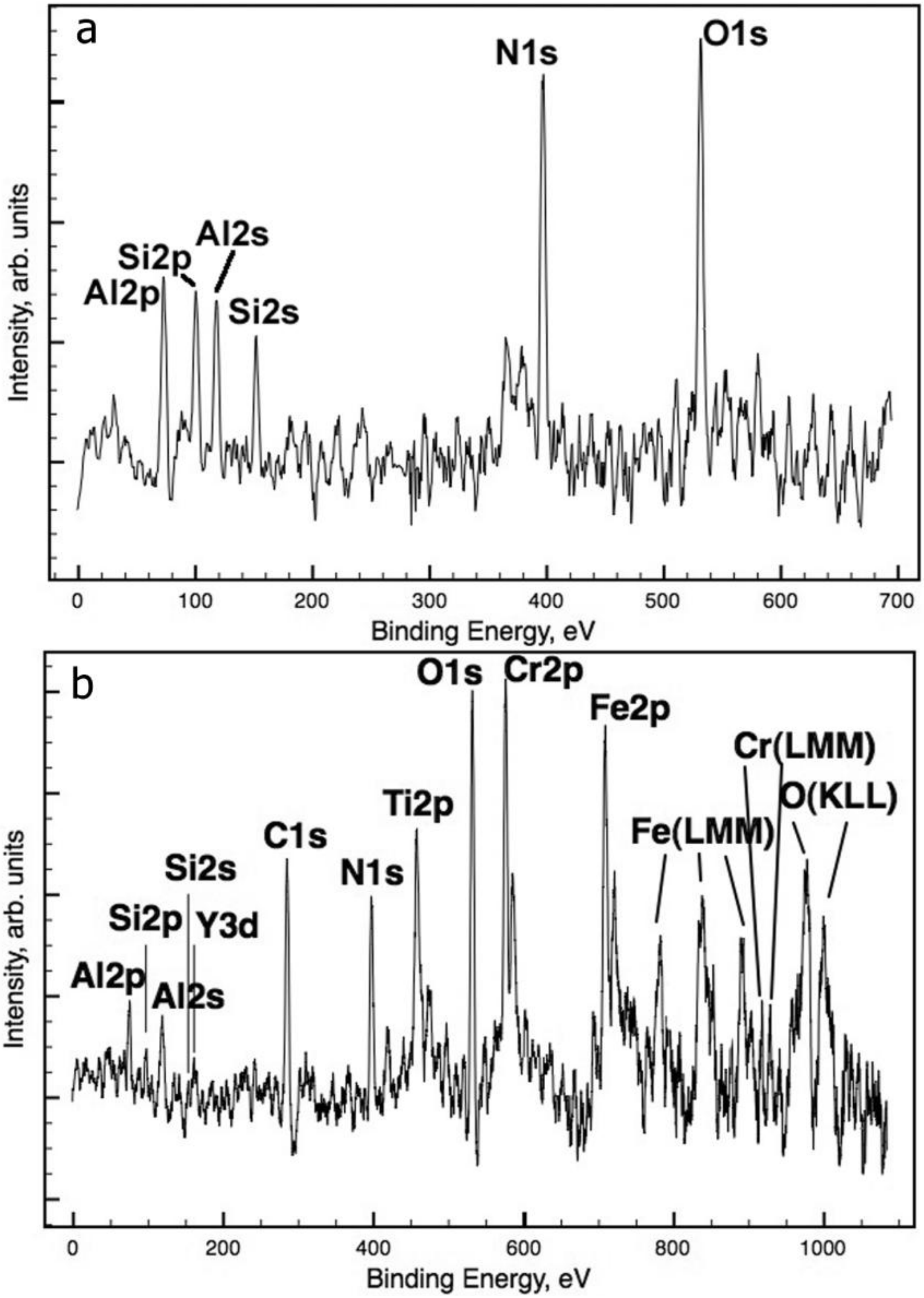
a
N1s
O1s
Si2p
Al2s
Al2p
Si2s
Intensity, arb. units
0
100
200
300
400
500
600
700
Binding Energy, eV
b
O1s
Cr2p
Fe2p
Cr(LMM)
Ti2p
O(KLL)
Fe(LMM)
C1s
Si2s
N1s
Si2p
Y3d
Al2p
Al2s
Intensity, arb. units
0
200
400
600
800
1000
Binding Energy, eV

.

Figure 2 SI

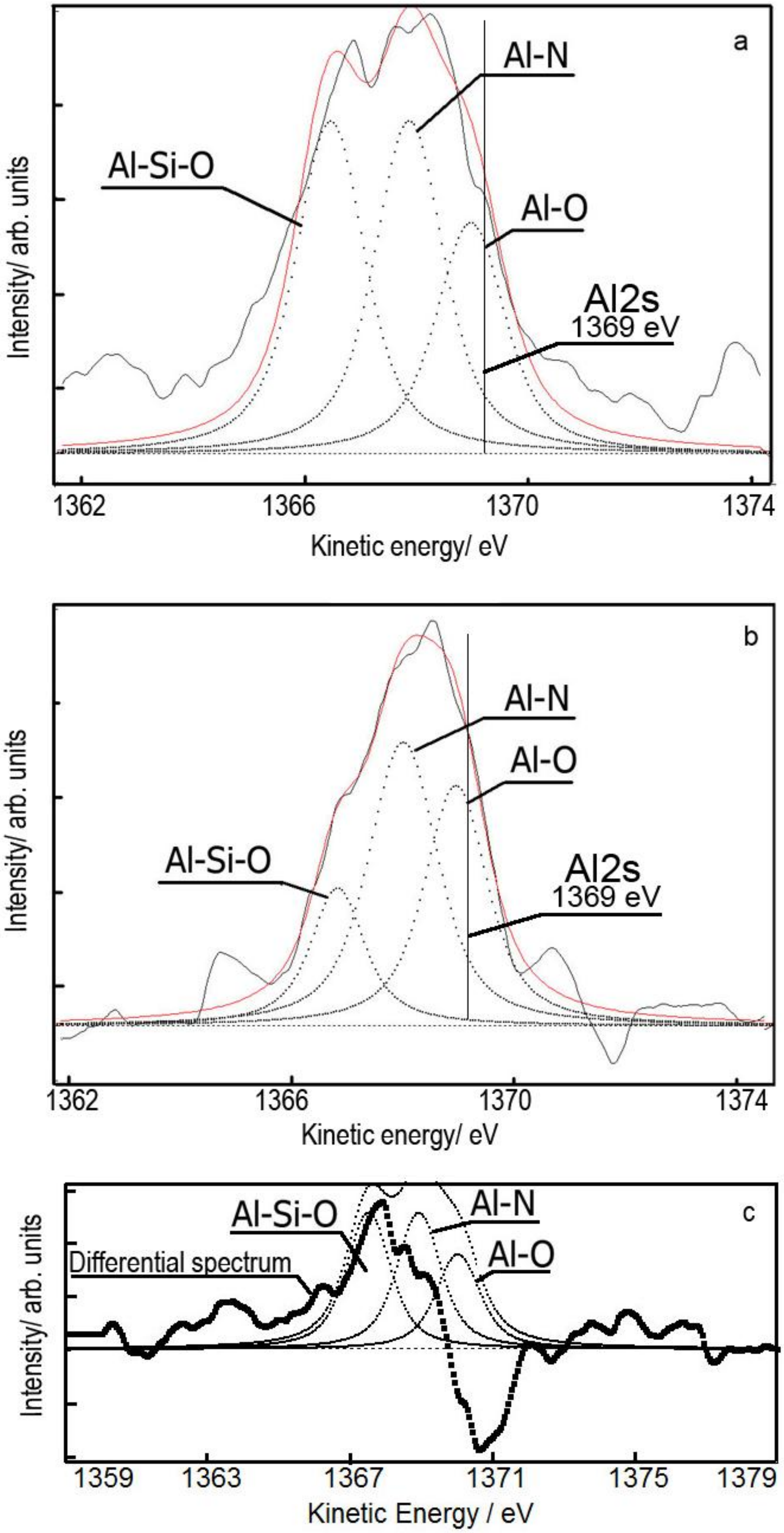
a
Al-N
Al-Si-O
Al-O
Al2s
1369 eV
Intensity/ arb. units
1362
1366
1370
1374
Kinetic energy/ eV
b
Al-N
Al-O
Al-Si-O
Al2s
1369 eV
Intensity/ arb. units
1362
1366
1370
1374
Kinetic energy/ eV
c
Al-Si-O
Al-N
Al-O
Differential spectrum
Intensity/ arb. units
1359
1363
1367
1371
1375
1379
Kinetic Energy / eV

Figure 3 SI

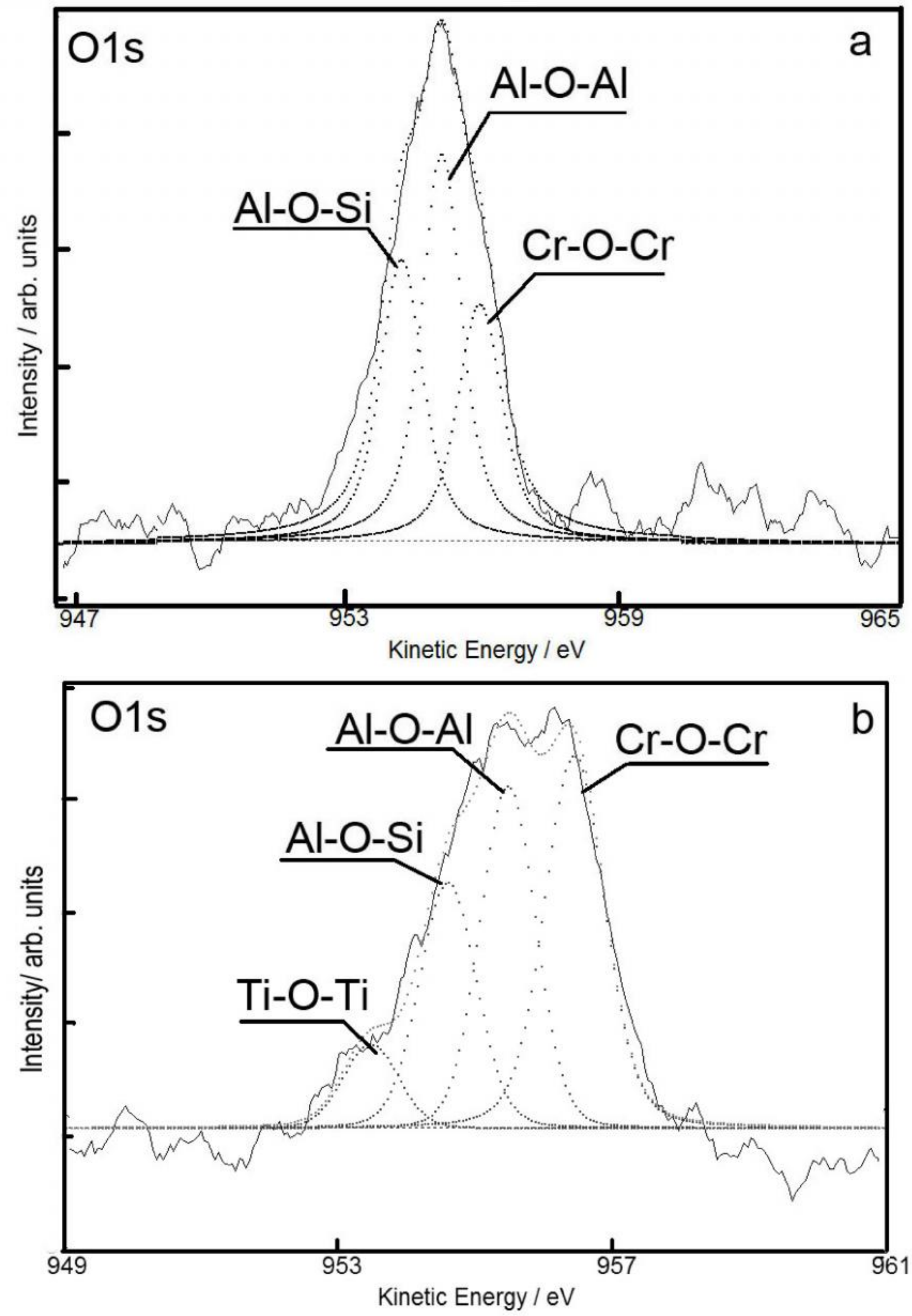
O1s
a
Al-O-Al
Al-O-Si
Cr-O-Cr
Intensity / arb. units
947
953
959
965
Kinetic Energy / eV
O1s
b
Al-O-Al
Cr-O-Cr
Al-O-Si
Ti-O-Ti
Intensity/ arb. units
949
953
957
961
Kinetic Energy / eV

Figure 4 SI

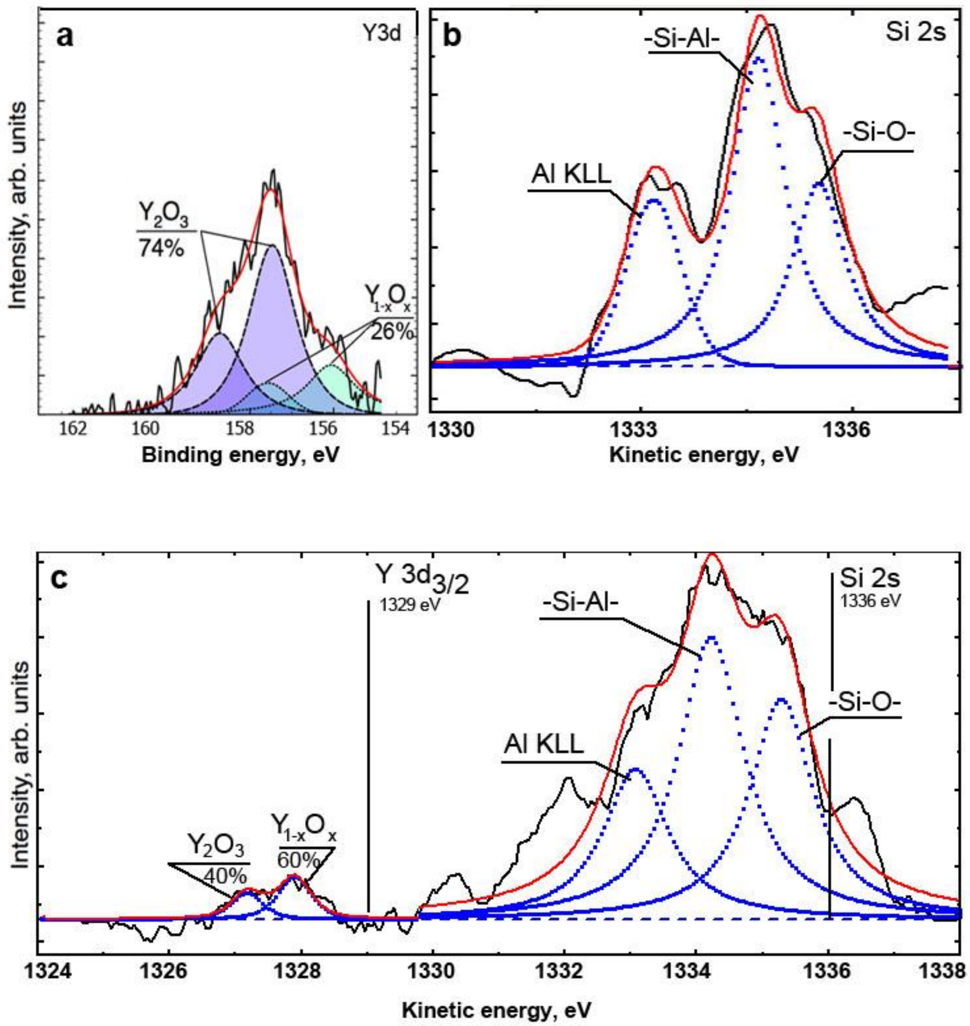

a
Y3d
Intensity, arb. units
Y_2O_3
74%
$Y_{1-x}O_x$
26%
162
160
158
156
154
Binding energy, eV
b
Si 2s
-Si-Al-
-Si-O-
Al KLL
1330
1333
1336
Kinetic energy, eV
c
Y $3d_{3/2}$
1329 eV
-Si-Al-
Si 2s
1336 eV
-Si-O-
Al KLL
Y_2O_3
40%
$Y_{1-x}O_x$
60%
Intensity, arb. units
1324
1326
1328
1330
1332
1334
1336
1338
Kinetic energy, eV
